# The Story of the Insane from Year to Year

**Published:** 1897-04-24

**Authors:** 


### THE STORY OF THE INSANE FROM YEAR TO YEAR.

Ninth Series.

### INVERNESS DISTRICT ASYLUM.

An increase from 471 to 486 has to be recorded during the year. There were 101 admissions, 56 readmissions, 86 recoveries, 21 discharged relieved, and 29 deaths. The percentage of recoveries on admissions was 54·7, but the number of readmissions was also very high, and this detracts from the value of a high recovery rate. The death-rate was only 6·3 per cent. on the average number resident. Of the deaths 8 were caused by cerebral diseases and 7 by consumption. Intemperance in drink accounts for 15 of the admissions, and hereditary influence for 62. When referring to the readmissions, Dr. Keay says that it is "to be accounted for by the efforts made in recent years to check the growth of the asylum population by boarding out chronic cases." There is no doubt that this result is obtained for a while, but it is only putting off the evil day, for it is, unhappily, too certain that the higher the discharge rate in this generation the greater will be the admission rate in the next. The low death-rate will show that the general health of the patients has been good, and Dr. Keay is fortunate in not having to chronicle any serious accident. The Commissioners say that " Dr. Keay spares no effort to provide for the patients in a thoroughly satisfactory manner."

### PERTH ROYAL ASYLUM.

On January 1st, 1895, this asylum contained 101 patients, and on the last day of the year the number was 108. There were 27 admissions, 12 readmissions, 14 recoveries, 12 discharged relieved, and 3 deaths. Calculated on the admissions, the recoveries stand at 35·90 per cent., and the deaths at 2·93 on the average number resident. Nine of the year's admissions owed their insanity to drink, and 18 to hereditary influence. When referring to the causes of insanity, Dr. Urquhart has the following sensible remarks: " It cannot be in the interests of humanity that habitual drunkards are permitted to work their own destruction and the ruin of their families without such legal restraints as are imposed upon other dangerous members of society," and he thinks the times are ripe for something to be done in the matter. We wish we could see any hope of legislation on the subject in the near future; but the hope grows dim within us. The ordinary minimum rate of board is £60 per annum, but we learn that during the last year 36 patients were maintained at from £30 to £52.

